# Supplementary material for: Clinical Impact of RANK Signalling in Ovarian Cancer
Source: Cancers (Basel). 2019 Jun 8;11(6):791. doi: 10.3390/cancers11060791 (PMC6627676; doi:10.3390/cancers11060791)
Supplement: Supplementary file 1 [file cancers-11-00791-s001.pdf]

Supplementary Materials: Clinical Impact of RANK Signalling in Ovarian Cancer

Verena Wieser, Susanne Sprung, Irina Tsibulak, Johannes Haybaeck, Hubert Hackl, Heidelinde Fiegl, Christian Marth and Alain Gustave Zeimet

Table S1. Association of *RANK*, *RANKL* and *OPG* mRNA expression with clinicopathological features in OC patients. *n* = 192.

All Tumours				*RANK* mRNA Expression (rel. to TBP)			*RANKL* mRNA Expression (rel. to TBP)			*OPG* mRNA Expression (rel. to TBP)		
**Variable**		***n***	**%**	**Median**	**IQR**	***p* Value**	**Median**	**IQR**	***p* Value**	**Median**	**IQR**	***p* Value**
Age	<50.0 yrs.	32	16.7	3.51	1.84–7.42	0.637	0.21	0.10–0.77	0.959	1.03	0.56–2.93	0.161
	≥50.0 yrs.	160	83.3	3.85	1.80–9.09		0.30	0.09–0.94		0.68	0.34–1.89	
FIGO stage	I	38	19.8	4.18	1.74–6.60	0.206	0.22	0.05–1.76	0.712	0.67	0.35–1.83	0.958
	II	12	6.3	3.66	1.99–5.94		0.15	0.07–0.81		0.60	0.34–2.91	
	III	121	63.0	3.39	1.63–5.60		0.30	0.09–0.81		0.80	0.32–2.13	
	IV	21	10.9	5.69	2.66–8.05		0.46	0.14–0.76		0.74	0.47–1.41	
Tumour grade	1	14	7.3	2.97	1.24–6.88	0.111	0.35	0.07–0.83	0.949	1.69	0.69–3.64	0.120
	2	85	44.3	3.12	1.49–6.20		0.29	0.10–1.03		0.66	0.33–2.10	
	3	91	47.4	4.14	2.24–6.48		0.30	0.09–0.90		0.75	0.38–1.79	
	unknown	2	1.0									
Residual disease After surgery	no	96	50.0	3.91	1.71–6.33	0.980	0.25	0.06–0.92	0.086	0.70	0.32–1.72	0.138
	yes	90	46.9	3.75	2.17–5.99		0.42	0.13–0.95		0.88	0.44–2.47	
	unknown	6	3.1									
Histology	HGSOC	122	63.5	3.25	1.68–5.84	**0.012**	0.29	0.10–0.77	0.432	0.63	0.37–1.78	0.112
	LGSOC	12	6.3	2.81	1.19–6.68		0.10	0.07–0.66		1.69	0.72–3.00	
	endometroid OC	44	22.9	4.00	2.00–8.05		0.32	0.09–1.94		0.81	0.31–2.65	
	clear cell OC	10	5.2	7.61	4.59–9.64		0.17	0.05–0.55		1.42	0.51–2.66	
	unknown	4	2.1									
BRCA1/2 mutation	no	146	76.0	3.73	1.77–6.24	0.646	0.30	0.09–0.73	**0.033**	0.65	0.33–0.84	0.107
	yes	44	22.9	4.00	1.82–6.53		0.55	0.18–1.08		1.19	0.38–3.14	
	unknown	2	1.0									

Table S2. IHC scoring of RANKL, RANK and OPG regarding their distribution to intra-tumour epithelial and stromal cells. Median tumour purity and median mRNA expression of *RANKL*, *RANK* and *OPG* of IHC specimens ($n = 20$).

Variable Title	Median	IQR
RANKL intra-tumour epithelial protein expression (% of positive cells)	90.0	90.0–90.0
RANKL intra-tumour stromal protein expression (% of positive cells)	80.0	65.0–90.0
RANK intra-tumour epithelial protein expression (% of positive cells)	90.0	80.0–99.0
RANK intra-tumour stromal protein expression (% of positive cells)	80.0	70.0–90.0
OPG intra-tumour epithelial protein expression (% of positive cells)	70.0	0.0–40.0
OPG intra-tumour stromal protein expression (% of positive cells)	15.0	5.0–80.1
Tumour purity (%)	75.0	60.0–82.5
RANKL mRNA expression (rel. to TBP)	0.07	0.03–0.49
RANK mRNA expression (rel. to TBP)	3.34	1.15–9.71
OPG mRNA expression (rel. to TBP)	0.87	0.49–1.15

Table S3. Spearman correlation analysis of tumour purity on IHC samples and RNA expression of *RANK*, *RANKL* and *OPG* ($n = 20$).

Variable		*RANKL* mRNA Expression (rel. to TBP)	*RANK* mRNA Expression (rel. to TBP)	*OPG* mRNA Expression (rel. to TBP)
Tumour Purity (%)	*rs*	−0.002	0.077	−.231
	p-Value	0.992	0.732	0.3011

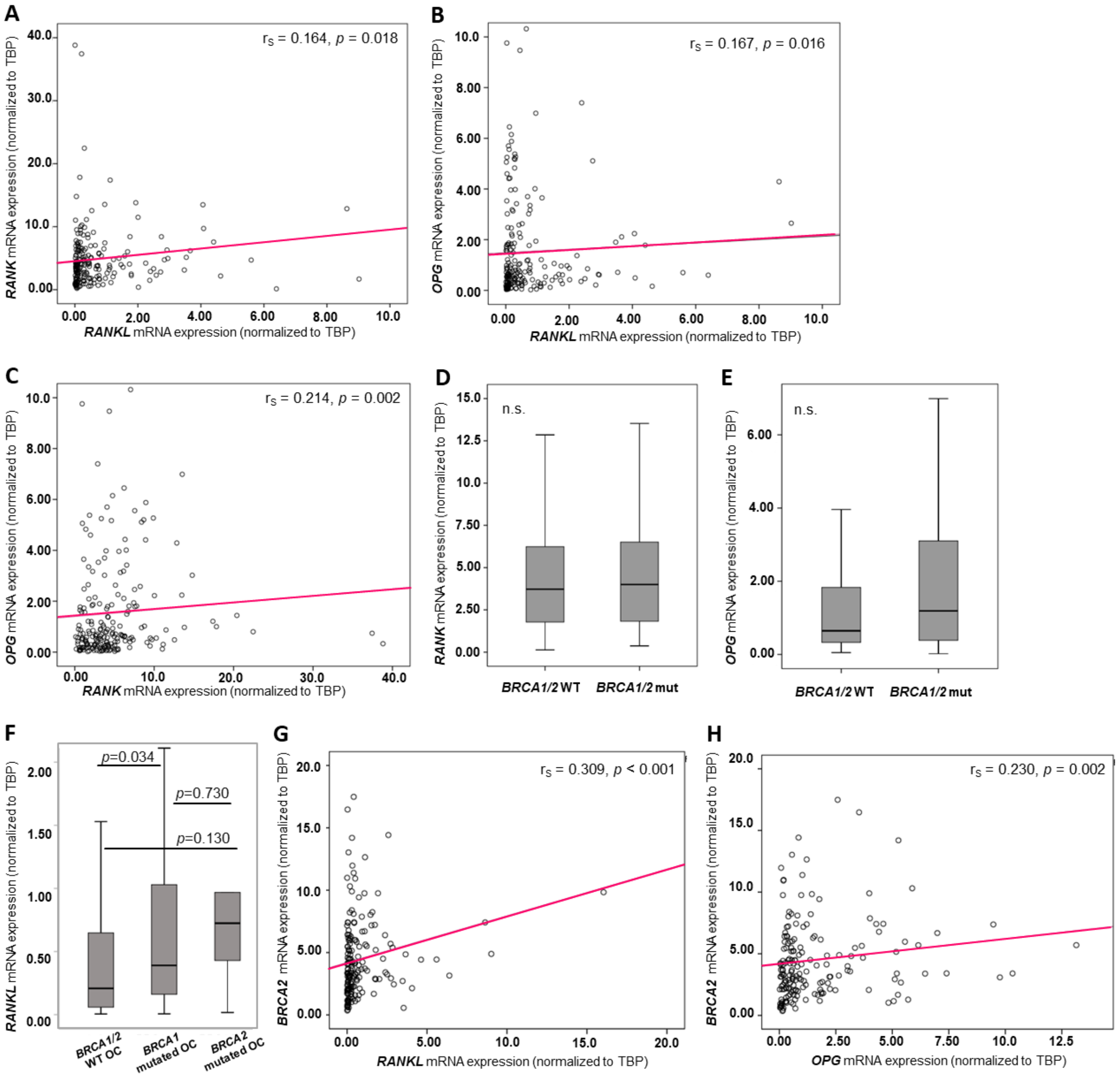

Figure S1. *RANK, RANKL and OPG* mRNA expression levels in OC, *BRCA1/2* mutated OC and correlation analyses. Linear regression analysis of (**A**) *RANK* and *RANKL*, (**B**) *OPG* and *RANKL* and (**C**) *OPG* and *RANK* in non-malignant tubes and OC (n = 206). (**D**) *RANK* and (**E**) *OPG* mRNA expression in *BRCA1/2* mutated OC (n = 44) compared to *BRCA1/2* wildtype (WT) tumours (n = 146). (**F**) *RANKL* mRNA expression in *BRCA1* mutated OC (n = 35), *BRCA2* mutated OC (n = 9) compared to *BRCA1/2* WT OC (n = 146). Linear regression analysis of (**G**) *BRCA2* and *RANKL* and (**H**) *BRCA2* and *OPG* in non-malignant tubes and OC (n = 206). *RANK, RANKL* and *OPG* mRNA expression values were normalized to *TBP* expression.

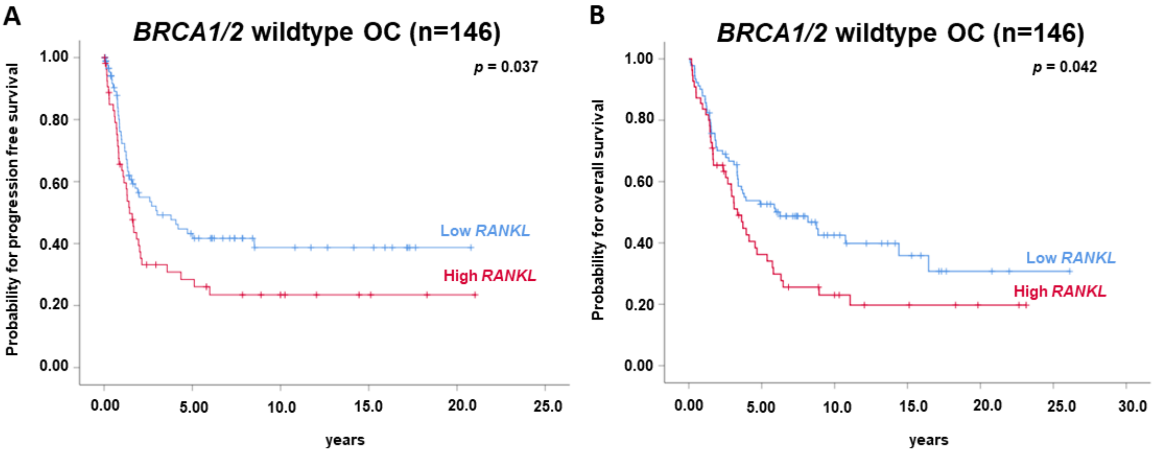

Figure S2. High *RANKL* mRNA expressions are associated with worse PFS and OS in the subgroup of patients with *BRCA1/2* wildtype tumours (n = 146). *RANKL* mRNA expression and (**A**) progression free survival and (**B**) overall survival. *RANKL* mRNA expression values were normalized to *TBP* expression.

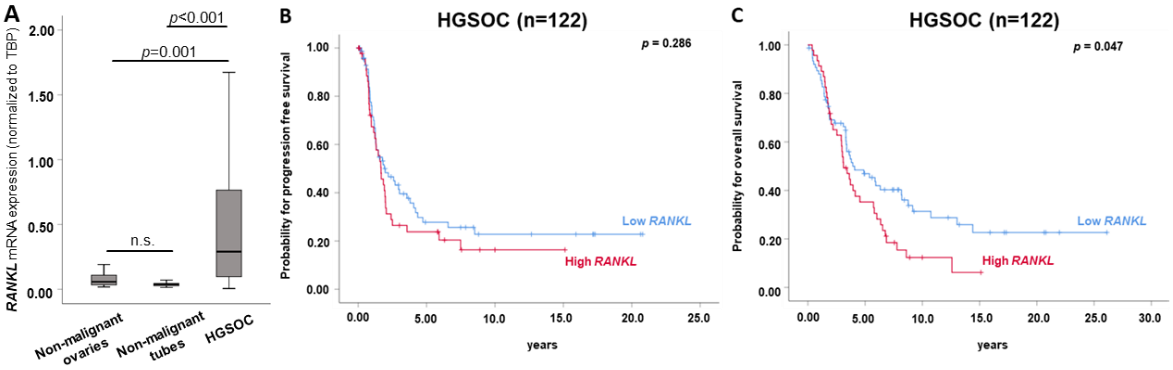

Figure S3. *RANKL* expressions are elevated in HGSOC compared to non-malignant ovaries and Fallopian tubes and associated with worse PFS and OS in the subgroup of HGSOC patients. (**A**) *RANKL* mRNA expression non-malignant ovaries (n = 21), non-malignant fallopian tubes (n = 14) and HGSOC (n = 122). *RANKL* mRNA expression in association with (**B**) progression free survival and (**C**) overall survival in HGSOC patients (n = 122). *RANKL* mRNA expression values were normalized to *TBP* expression.

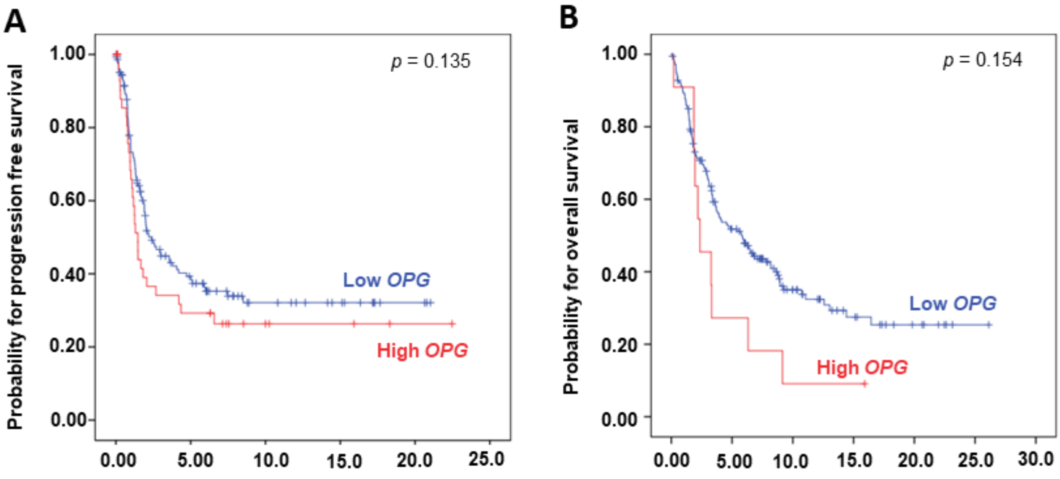

Figure S4. Kaplan—Meier survival analyses of *OPG* mRNA-expression in OC patients. *OPG* mRNA expression (n = 192) and (**A**) progression free survival and (**B**) overall survival. *OPG* mRNA expression values were normalized to *TBP* expression.

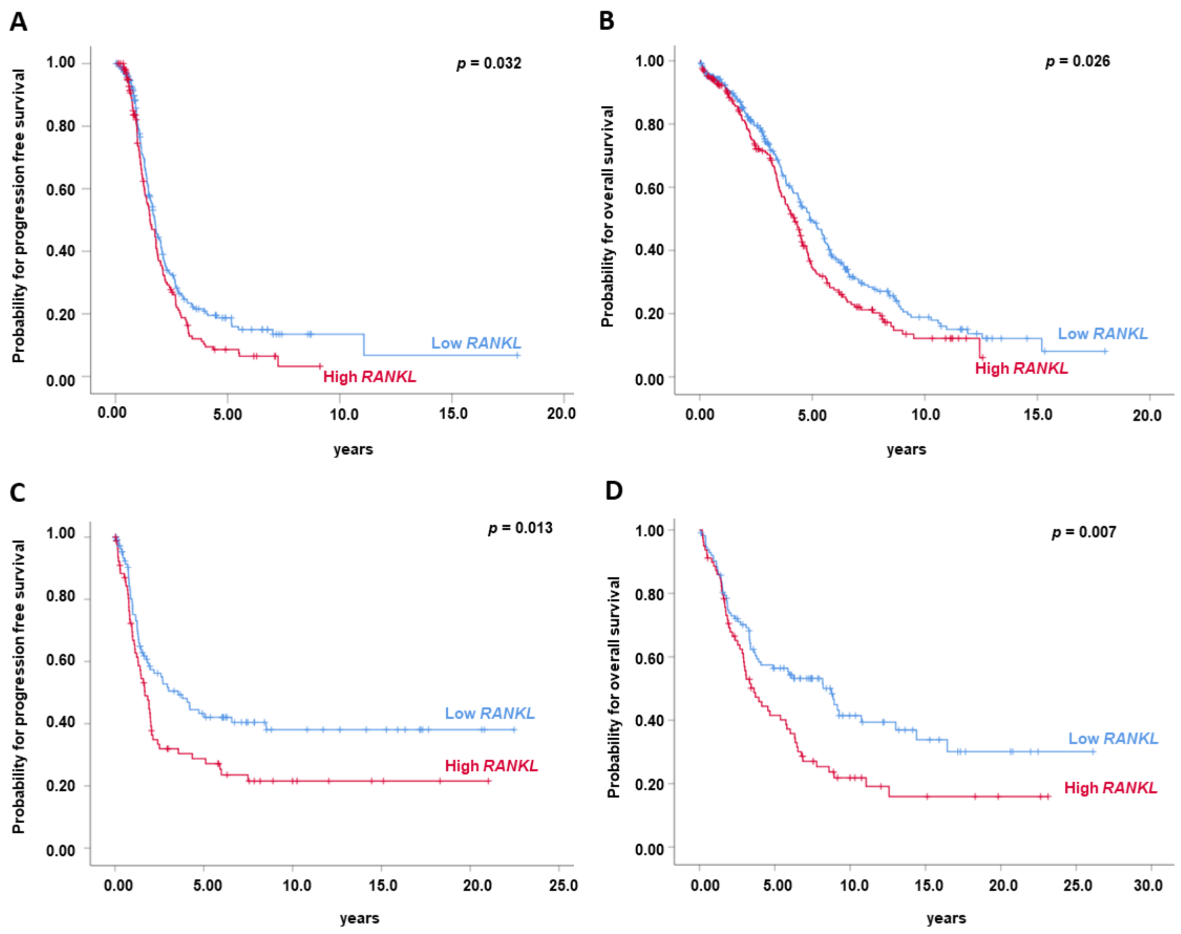

Figure S5. Kaplan—Meier survival analyses of *RANKL* mRNA-expression with optimal cut-offs determined in the TCGA cohort. (**A**,**B**) *RANKL* mRNA expression in the TCGA dataset with optimal cut-off determined by Youden-Index in association with progression free survival (**A**) and overall survival (**B**). (**C**,**D**) *RANKL* mRNA expression in our cohort (n = 192) (with the cut-off determined for the TCGA cohort) in association with (**C**) progression free survival and (**D**) overall survival. *RANKL* mRNA expression values were normalized to *TBP* expression.

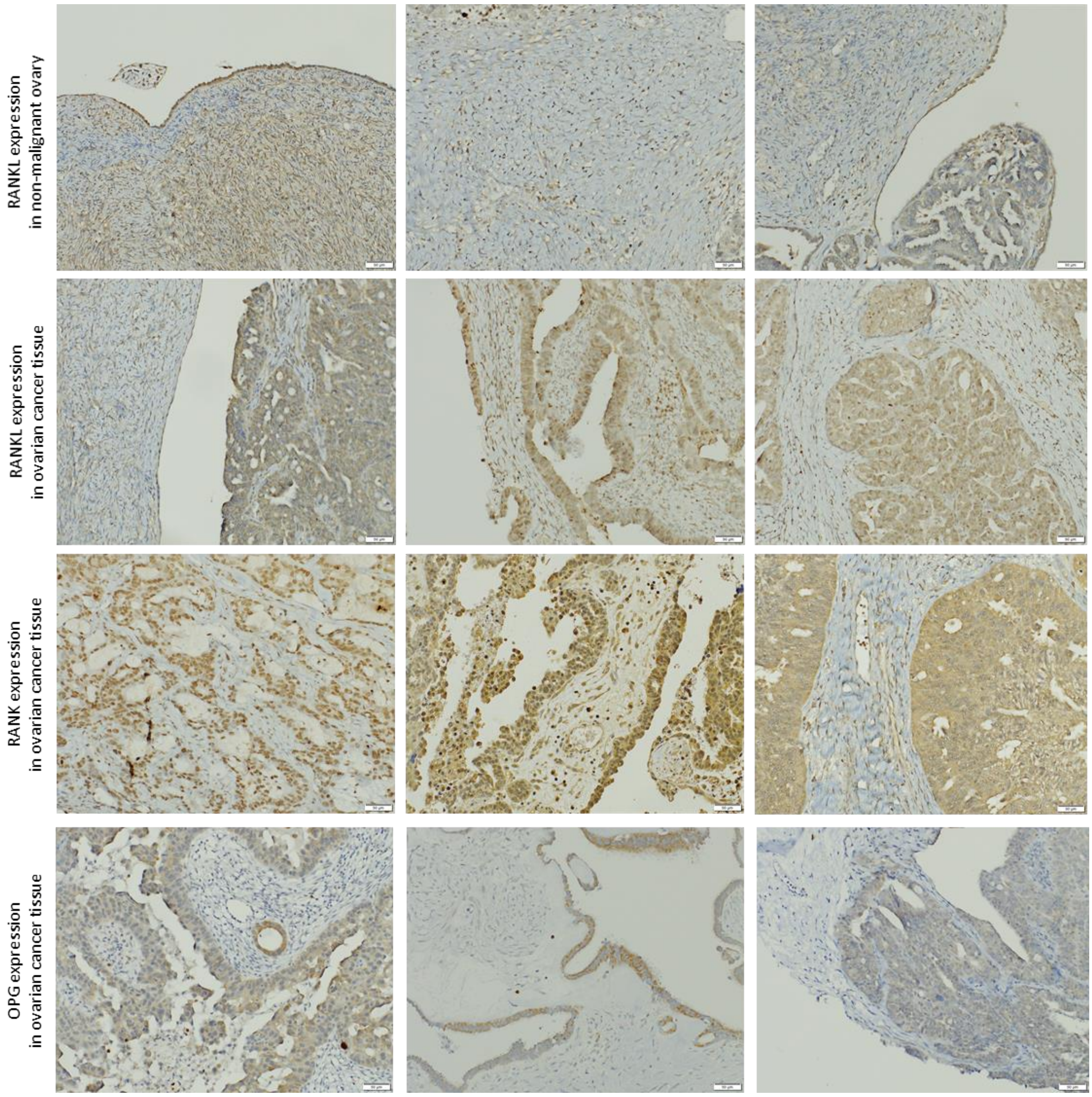

Figure S6. RANK, RANKL and OPG localize to cancer cells and tumour microenvironment in OC. Representative RANK, RANKL and OPG immunohistochemistry on FFPE sections from non-malignant ovaries and ovarian cancer tissues. Scale bars indicate 50 μm.

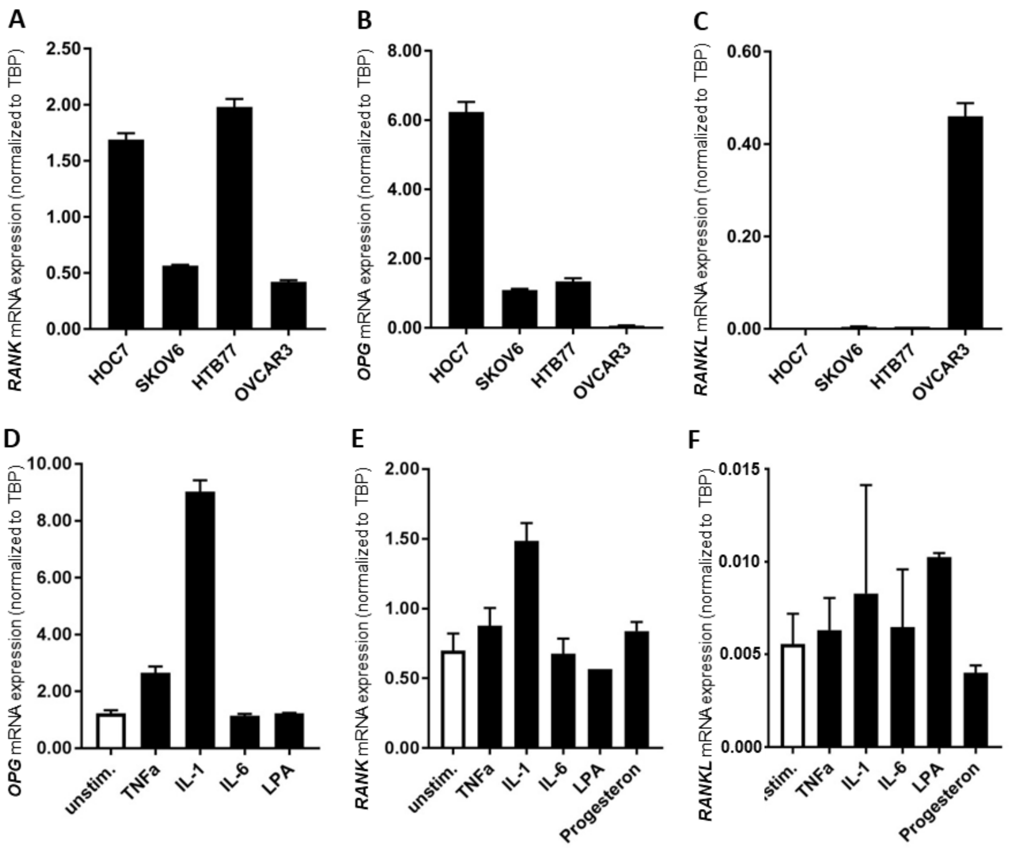

Figure S7. ***R****ANK*/*RANKL*/*OPG* are expressed in the human OC cell lines HOC7, SKOV6, HTB77 and OVCAR3 whereas *RANK* and *OPG* can be induced by inflammatory stimuli in OC cell lines. Baseline (**A**) *RANK* (**B**) *OPG* and (**C**) *RANKL* expression. (**D**) *OPG*, (**E**) *RANK* in SKOV6 after stimulation with TNFα, IL-1β, IL-6 and LPA for 6 hours (n = 3). (**F**) *RANKL* expression in SKOV6 after stimulation with TNFα, IL-1β, IL-6, LPA and progesterone for 6 hours (n = 3). *RANK, RANKL* and *OPG* mRNA expression values were normalized to *TBP* expression.

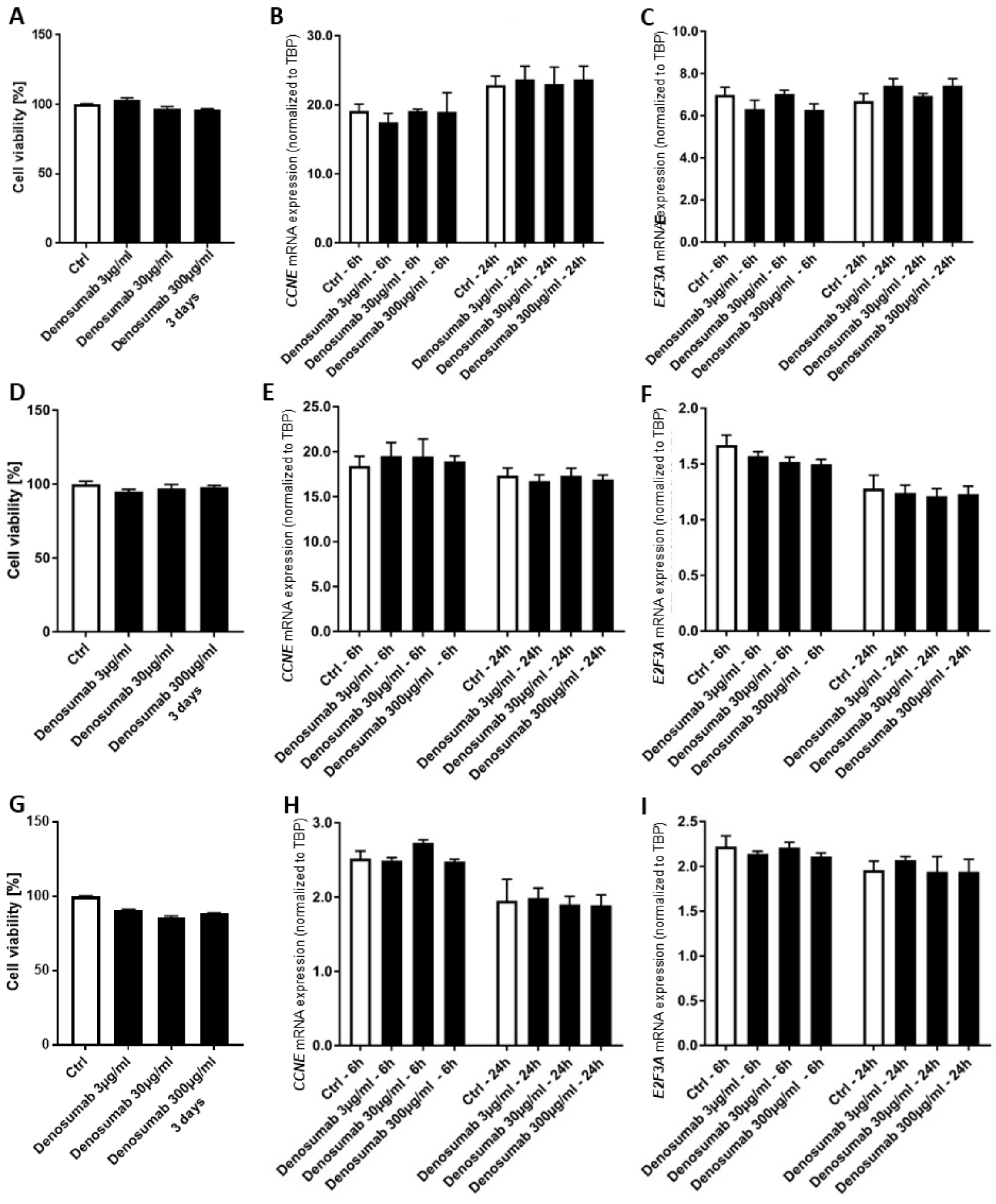

Figure S8. Blocking RANK/RANKL signalling using denosumab influenced neither OC cell viability nor cell cycle regulation. (**A**) Cell viability (**B**) *CCNE* and (**C**) *E2F3A* expression after denosumab treatment with indicated concentrations in OVCAR3 ($n = 3$). (**D**) Cell viability (**E**) *CCNE* and (**F**) *E2F3A* expression after denosumab treatment with indicated concentrations in SKOV6 ($n = 3$). (**G**) Cell viability (**H**) *CCNE* and (**I**) *E2F3A* expression after denosumab treatment with indicated concentrations in HTB77 ($n = 3$). Viability was assessed after 3 days denosumab treatment by MTT test. *CCNE* and *E2F3A* mRNA expression values were normalized to *TBP* expression.

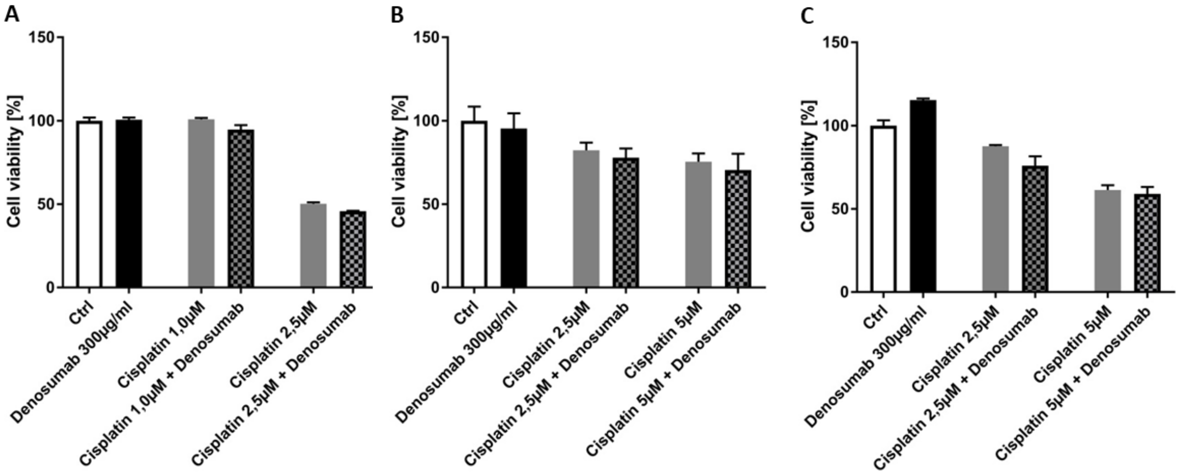

Figure S9. Blocking RANK/RANKL signalling using denosumab did not influence platinum-induced OC cell toxicity. Cell viability after cisplatin +/− denosumab treatment for 3 days in (**A**) OVCAR3, (**B**) SKOV6 and (**C**) HTB77 (n = 3, respectively). Viability was assessed after treatment with cisplatin +/− denosumab by MTT test.

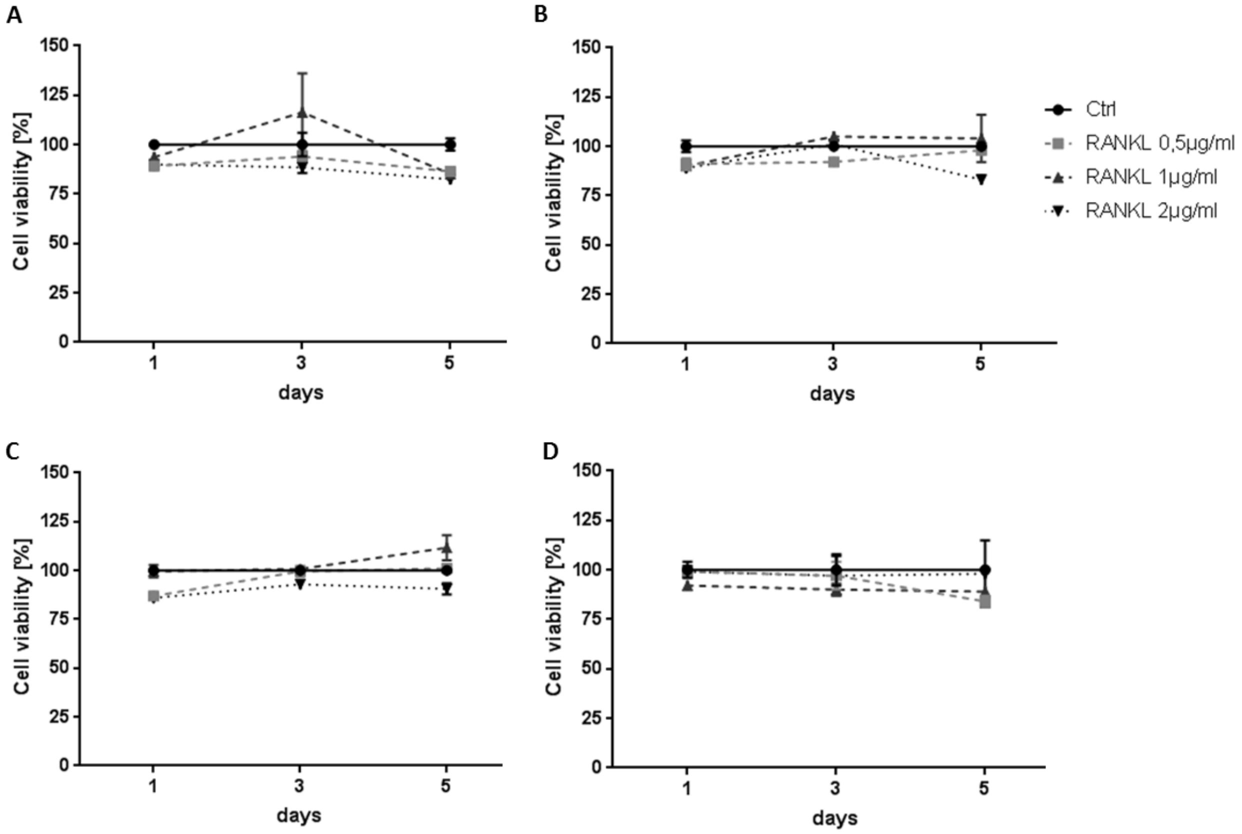

Figure S10. Recombinant RANKL did not influence platinum-induced OC viability. Time scores of cell viability after RANKL treatment in (**A**) OVCAR3, (**B**) SKOV6 and (**C**) HTB77and (**D**) HOC7 cell lines. Viability was assessed at indicated time points by MTT test.

© 2019 by the authors. Licensee MDPI, Basel, Switzerland. This article is an open access article distributed under the terms and conditions of the Creative Commons Attribution (CC BY) license (http://creativecommons.org/licenses/by/4.0/).
